# Inward Tension of Talin and Integrin-related Osmotic Pressure are involved Synergetically in the Invasion and Metastasis of Non-small Cell Lung Cancer

**DOI:** 10.7150/jca.45494

**Published:** 2020-06-23

**Authors:** Ying Song, Chen Li, Yahan Fu, Qiu Xie, Jun Guo, Guangming Li, Huiwen Wu

**Affiliations:** 1 Department of Respiratory Medicine, The First Affiliated Hospital of Nanjing Medical University, Nanjing 210029, PR China.; 2 School of Medicine & Holistic Integrative Medicine, Nanjing University of Chinese Medicine, Nanjing 210023, PR China.; 3 Department of Anesthesiology, Huaian First People's Hospital, Nanjing Medical University, Huaian 223001, PR China.; 4 Laboratory Center for Basic Medical Sciences, Nanjing Medical University, Nanjing 211166, PR China.

**Keywords:** integrin receptor, non-small cell lung cancer, invasion, metastasis

## Abstract

The integrin receptor protein talin plays vital roles in intracellular chemical and mechanical activities, and it is implicated in the high invasion and poor prognosis of non-small cell lung cancer (NSCLC). To better understand the mechanism underlying the function of talin in NSCLC invasion and metastasis, a few newly designed tension probe based on Förster resonance energy transfer was used for real-time observation of tension changes in A549 cells. High NSCLC cell aggressiveness was found to be accompanied with inward talin and outward glial fibrillary acidic protein (GFAP) tensions, which are closely associated with microfilament (MF) force and intracellular osmotic potential. The increased osmotic pressure resulted from the production of intracellular protein nanoparticles and the related ion influx. Furthermore, integrin activation was found to adjust the talin and GFAP tensions. Disruption of the interaction between talin and MFs blocked the mechanical source of talin, reducing both talin tension and osmotic pressure and thus inhibiting NSCLC cell invasion and migration. Consequently, our study demonstrates that talin is involved in NSCLC invasion and migration via its inward tension and that the integrin pathway is correlated closely with protein-nanoparticle-induced outward osmotic pressure.

## Introduction

Lung cancer is the leading cause of cancer mortality worldwide in both men and women, and metastases are considered to be the major cause of lung-cancer-related deaths 1. Non-small cell lung cancer (NSCLC) is the most common subtype of lung cancer, and its liver metastasis is a major reason for poor NSCLC prognoses 2, 3. Numerous studies suggested that tumor migration depends on intracellular mechanical activities 3, 4. Therefore, there is an urgent need to elucidate the mechanical mechanisms underlying invasion and metastasis in NSCLC.

The cytoskeletal protein talin is widely implicated in cancer progression processes, such as cell polarization, migration, and metastasis 4-7. It contains an N-terminal head domain and a large C-terminal flexible rod domain 8, 9. The talin head can directly bind to integrin β subunit cytoplasmic domains, activating them and strengthening their interaction with extracellular matrix proteins 10-14. The association between integrin and the extracellular matrix leads to the formation of focal adhesions (FAs) and cell adhesion, which are considered to be of fundamental importance for tumor migration 15. Furthermore, talin contains three actin-binding sites (ABSs), with ABS3 in the rod domain, generally thought to be the most important for interaction between talin and microfilaments (MFs) 8, 16. The C-terminal actin binding site is necessary for invadopodium maturation and actin polymerization equilibrium at invadopodia 17, 18.

Previous studies have demonstrated that the talin complex is a vital signaling scaffold in the transmission of chemical and mechanical signals in cellular physiological and pathological activities 10, 19-21. The talin-integrin complex transmits force dynamically and induces matrix degradation in FAs near cell edges. Furthermore, talin has been found to localize at invadopodia and to regulate cofilin-mediated actin depolymerization 15. MF depolymerization increases the production of protein nanoparticles and osmotic pressure 22, thus promoting cell migration. However, whether this mechanotransduction in NSCLC invasion and metastasis is mediated by interactions between MFs and talin and/or integrin activity remains to be elucidated.

Accordingly, in the present study, we investigated the relationship between talin-related tension and the aggressiveness of NSCLC cells. A newly designed tension probe based on Förster resonance energy transfer (FRET) was used for real-time monitoring of talin and glial fibrillary acidic protein (GFAP) tensions in A549 cells. Thus, we were able to ascertain the direction and magnitude of talin tension and osmotic pressure and analyze the mechanism underlying NSCLC invasion and migration.

## Methods

### Cell Culture

A549 cells were purchased from Shanghai Cell Bank Type Culture Collection Committee (CBTCCC, Shanghai, China). Cells were cultured in Dulbecco's Modified Eagle's Medium (Gibco, New York, USA) containing 10% fetal bovine serum (FBS, Gibco, New York, USA) and antibiotics (100 U/mL streptomycin and penicillin) under humidified air containing 5% CO_2_ at 37 °C.

### Reagents and Antibodies

Antibody against α-tubulin (11224-1-AP, Western blotting 1:5000, immunofluorescence 1:200) was obtained from Proteintech Group (Rosemont, IL, USA). Antibody against talin (A02859-1, Western blotting 1:500) was purchased from Boster Bio (Pleasanton, CA, USA). Fluorescein-conjugated goat anti-mouse IgG (H+L) (ZF-0312, IF 1:100) and fluorescein-conjugated goat anti-rabbit IgG (H+L) (ZF-0311, IF 1:100) were obtained from Zsgb-Bio (Beijing, China).

Ciliobrevin D, blebbistatin, and cilengitide were purchased from Aladdin (Shanghai, China). Ispinesib (SB-715992) was purchased from Beyotime Biotechnology (Jiangsu, China). Cytochalasin D (Cyto D) and nocodazole (Noc) were obtained from MilliporeSima (Burlington, MA, USA). Manganese chloride was obtained from Sinopharm (Beijing, China).

### Osmotic pressure measurement

Osmotic pressure was measured as reported previously 21, 22. Namely, the cells were broken using an ultrasonic crusher for 1min (VCX150; Sonics, Newtown, CT, USA), and then centrifuged at 13,000 g for 10 min at 4 °C. 50 μl of the supernatant solution (cytoplasm) was placed into 0.5-mL test tubes. An Osmomat 3000 Freezing Point Osmometer (Gonotec, Berlin, Germany) was calibrated thrice before use. Then, cytoplasmic OP was recorded.

### Western blotting

A549 cells were dissolved in radioimmunoprecipitation assay lysis buffer (Beyotime Bio) supplemented with phenylmethylsulfonyl fluoride (Roche, Basel, Switzerland) and a protease inhibitor cocktail (MilliporeSigma). Cells lysates were denatured by boiling in loading buffer. The extracted total proteins were separated using SDS-PAGE and transferred to nitrocellulose membranes, which were blocked by incubation in 5% nonfat milk for 1 h. The membranes were then incubated with specific antibodies overnight at 4 °C. After washing three times, the membranes were incubated with secondary antibodies for 2 h. Enhanced chemiluminescence (ECL) chromogenic substrate was used to visualize the immunoreactive protein bands, the intensities of which were quantified by densitometry (Quantity One; Bio-Rad, Hercules, CA, USA). Actin or tubulin were used as the control.

### Transwell assays

The upper chamber of a transwell apparatus (Corning, New York, USA) was pre-coated with 50 μL of matrigel solution. A549 cells (2 × 10^5^) were starved overnight and seeded into the upper chamber in serum-free medium. Then, medium supplemented with 20% FBS was added to the bottom chamber. After 24 h incubation, the cells were stained with 4% paraformaldehyde and dyed with crystal violet. Typical images of invading cells were obtained for statistical analysis. Migration assays were performed in a similar manner to the invasion assays, but without pre-coating with matrigel.

### cpstFRET analysis

The effectiveness of FRET in the stable monoclonal cell line was determined by the dipole angle between the donor (eCFP) and the acceptor (eYFP). Thereafter, the FRET analysis was conducted as reported in previous works 20-22. We calculated the CPF/FRET ratio, which is negatively correlated with FRET efficiency and positively correlated with force, using the relationship E = eCFP donor/eYFP acceptor.

### Acquisition of clinical samples and bioinformatics analysis

Fresh breast cancer tissue samples and adjacent normal tissues were obtained surgically from the Department of Surgical Oncology, The First Affiliated Hospital of Nanjing Medical University (Nanjing, China). All patients gave informed consent, and the study was ethically approved by the Institutional Review Board of Human Research of the Affiliated Hospital of Nanjing Medical University. The dates for survival analysis were obtained from the Kaplan-Meier plotter database. All patients were sorted into different groups and tested for significance using the log-rank test.

### Statistical analysis

Data are shown as mean ± SEM and verified using a Student's t test. One-way ANOVA was conducted using SPSS v.22.0 (IBM, Armonk, NY, USA) for single-factor sample comparisons, and a least significant difference test was used for comparisons between any two means. Each experiment was repeated at least three times, >10 cells were imaged, and each condition was analyzed.

## Results

### Talin-1 rather than talin-2 is associated with malignancy in NSCLC

**Figure 1:**
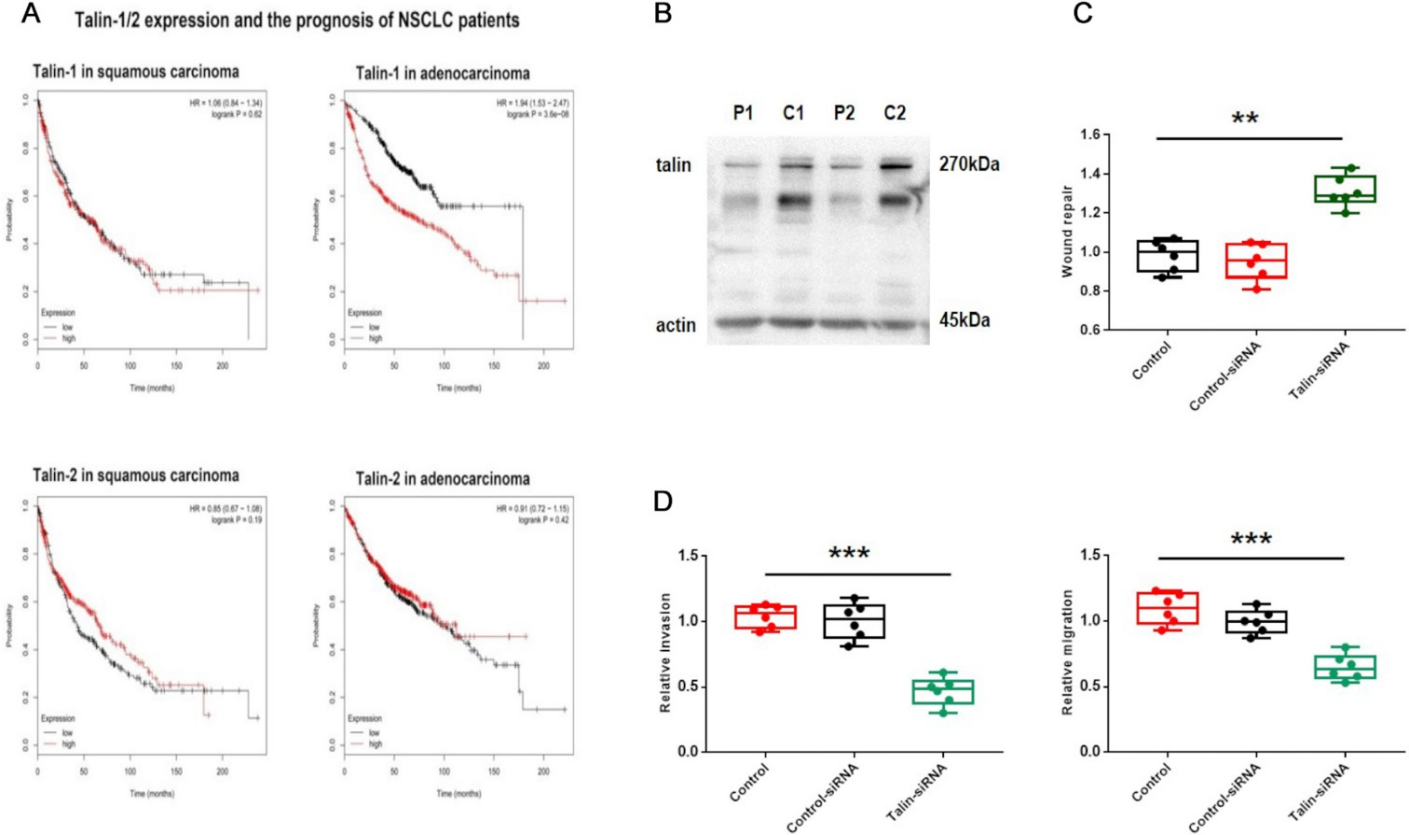
** Talin-1 rather than talin-2 is associated with malignancy in NSCLC** (**A**) Kaplan-Meier plots of overall survival of patients with squamous carcinoma and adenocarcinoma, stratified by talin-1 and talin-2 expression. (**B**) Western blotting of talin protein levels in fresh NSCLC tissue (**C**) and adjacent normal tissue (P). (C) Normalized quantitative analysis of the wound closure rate in A549 cells transfected with control siRNA or talin-1 siRNA. (**D**) Quantitative analysis of the migration and invasion rates. **P <0.01 compared with the control group, ***P <0.001 compared with the control group.

The integrin-binding adaptor protein talin is considered to be a regulator in tumor progression 4, 22, 23. Accordingly, to investigate the association between talin and NSCLC prognosis, the Kaplan-Meier plotter database was searched for relevant clinical data. The search revealed that lung adenocarcinoma patients with higher talin-1 expression show poorer overall survival than those with lower talin-1 expression (Fig. 1A). Furthermore, overall survival is not significantly different in lung squamous carcinoma patients with different talin-1 expression levels. However, talin-2 has no significant effect on prognosis in either lung squamous carcinoma or adenocarcinoma patients (Fig. 1A). These results indicate that high talin-1 expression rather than talin-2 expression leads to poor prognosis in lung adenocarcinoma patients.

Then, we analyzed the expression of talin-1 in patients with NSCLC (Fig. 1B). The expression of talin-1 in NSCLC samples is significantly higher than that in para-carcinoma tissues. Moreover, transwell and wound healing assays (Fig. 1) indicated that A549 cell aggressiveness is restrained after downregulation of talin-1. Thus, the data indicate that talin-1 instead of talin-2 is a positive regulator of NSCLC invasion and metastasis.

### Talin involvement in NSCLC invasion is correlated with MF force

**Figure 2:**
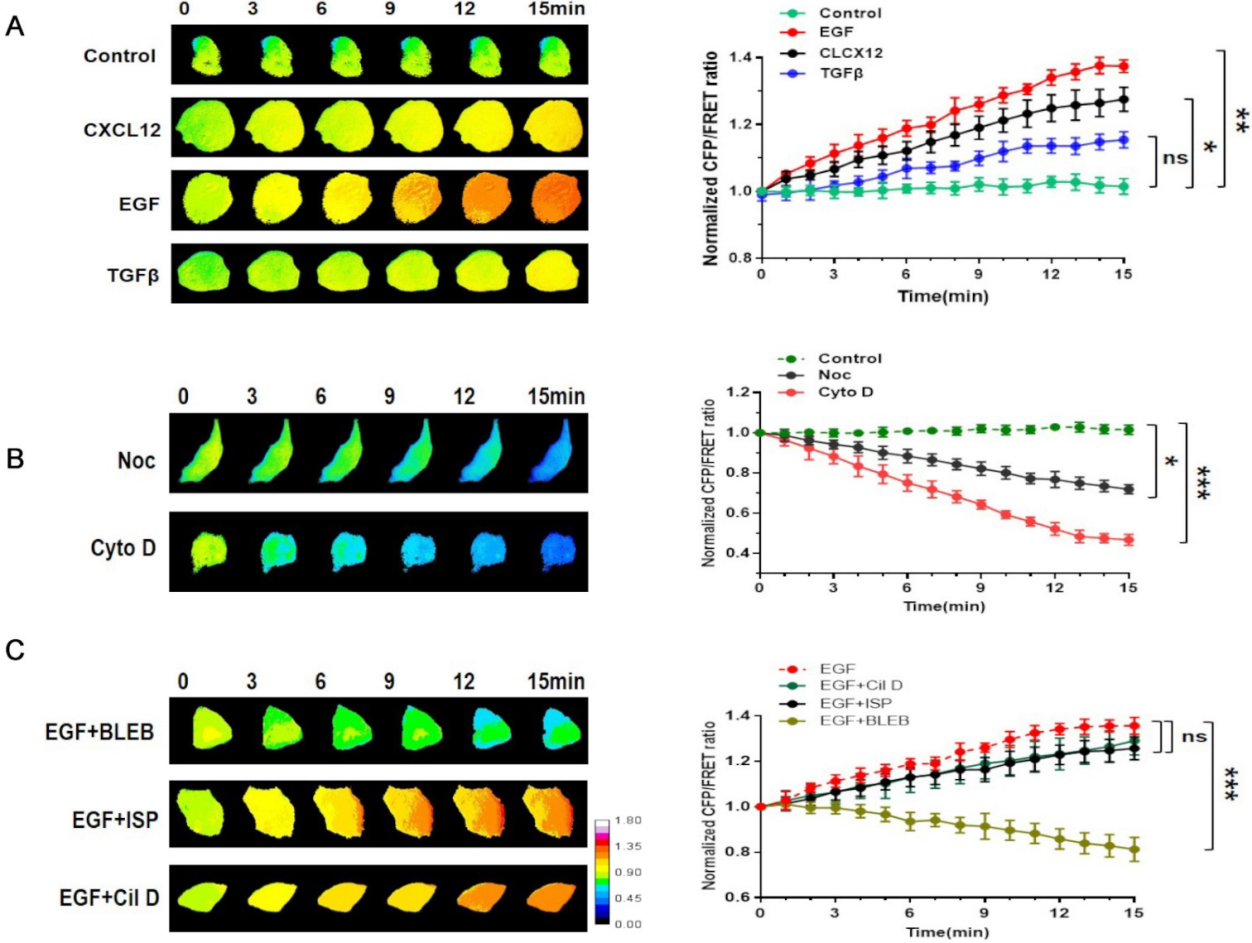
** Involvement of talin in NSCLC invasion is correlated with MF force** (**A**) Left panel: 15-min time-lapse images of FRET analysis in A549 cells expressing the talin-M-cpstFRET probe after CXCL12, EGF, or TGFβ treatments. Right panel: Normalized CFP and FRET signals corresponding to talin tension versus time. (**B**) Left panel: 15-min time-lapse images of FRET analysis in A549 cells expressing the talin-M-cpstFRET probe after Noc or Cyto D treatments. Right panel: Normalized CFP and FRET signals corresponding to talin tension versus time. (**C**) Left panel: 15-min time-lapse images of FRET analysis in A549 cells expressing the talin-M-cpstFRET probe after treatment with EGF in association with blebbistatin, ciliobrevin D, or ispinesib. Right panel: Normalized CFP and FRET signals corresponding to talin tension versus time (mean ± SEM, n ≥ 3).

Intracellular tension activity plays a crucial role in tumor invasion and migration 24. Accordingly, we used a talin-1 tension probe based on Förster resonance energy transfer (FRET), as reported in previous works 20-21, 25-27, to investigate the mechanical mechanism underlying the role of talin-1 in NSCLC cell aggressiveness. In order to establish a connection between talin tension and invasion/metastasis by NSCLC cells, we used three different stimulating factors, i.e., EGF, TGFβ, and CXCL12, to build short-term aggressive models. As shown in Fig. 2A, the results indicate that talin tension is positively correlated with A549 cell aggressiveness.

Then, the MF depolymerizing agent Cyto D and microtubule (MT) depolymerizing agent Noc were used to identify the mechanical source of talin tension. Talin tension shows an obvious decrease in A549 cells treated with Cyto D or Noc, with Cyto D treatment causing a sharper reduction.

Motor protein inhibitors 28 were used to monitor the source of talin tension (Fig. 2B). The myosin inhibitor blebbistatin clearly decreases talin tension, whereas the influences of the dynein inhibitor ciliobrevin and the kinesin inhibitor ispinesib on talin tension are not obvious (Fig. 2C). Thus, these results indicate that the increase in talin tension in NSCLC invasion and metastasis results from inward MF force.

### Epidermal growth factor (EGF) induces NSCLC invasion and migration via protein-nanoparticle-induced osmotic pressure

Osmotic pressure, especially protein-nanoparticle-induced osmotic pressure, may play an important role in the tension activity related to cancer invasion and metastasis 29. Accordingly, to investigate this phenomenon, we exploited to properties of GFAP, which is an intermediate filament protein that is highly sensitive to osmotic pressure changes in cells 22.

**Figure 3:**
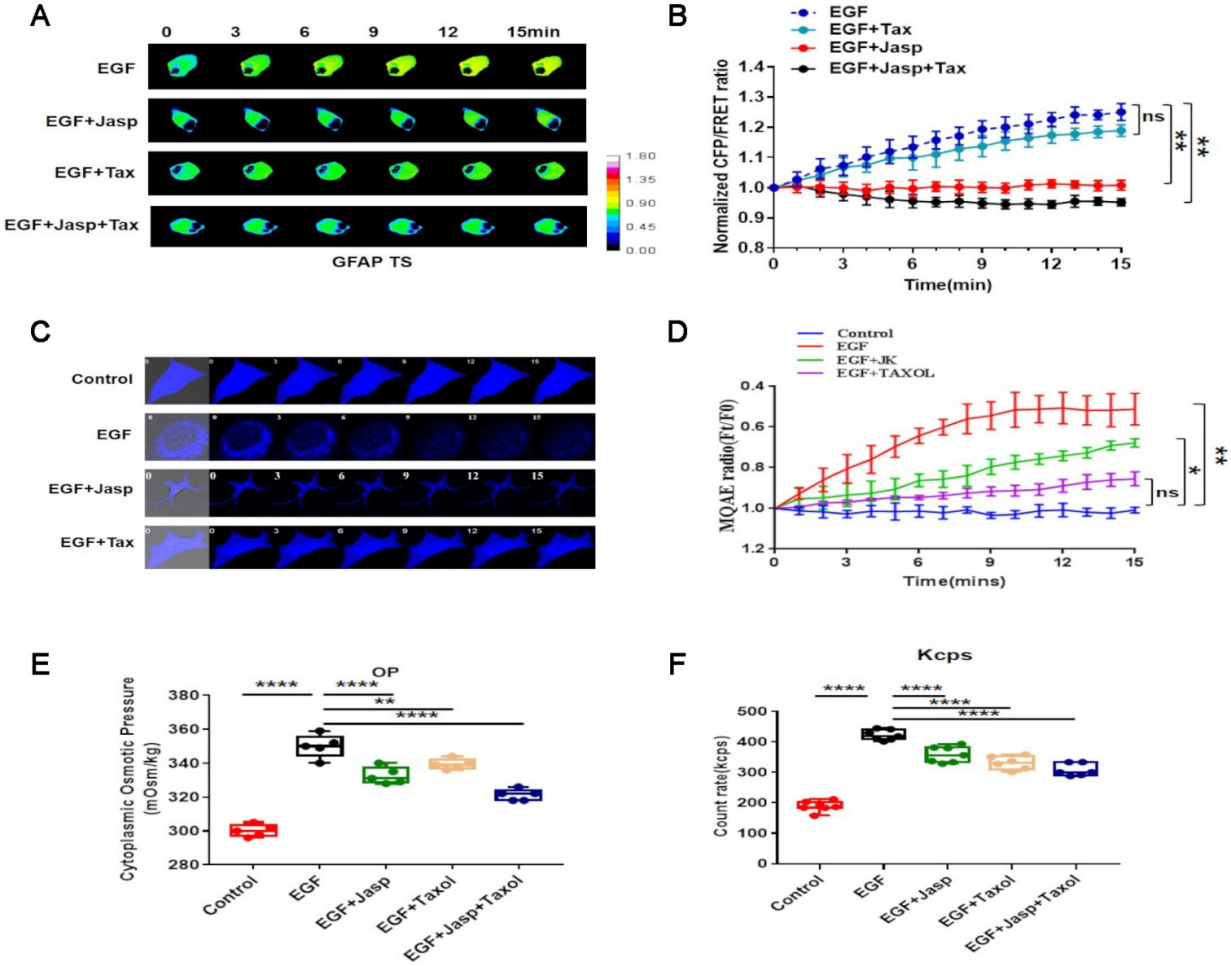
** EGF promotes protein-nanoparticle-induced osmotic pressure** (**A**) 15-min time-lapse images of FRET analysis in A549 cells expressing the GFAP-cpstFRET-GFAP probe after EGF, EGF+Jasp, EGF+Tax or EGF+Jasp+Tax treatment. (**B**) Normalized CFP and FRET signals corresponding to GFAP tension versus time. (**C**) 15-min time-lapse images of MQAE analysis after EGF, EGF+Jasp, or EGF+Jasp+Tax treatment in A549 cells. (**D**) Means of normalized MQAE fluorescence intensities. (**E**) Osmolalities of the control group, EGF, EGF+Jasp, EGF+Tax and EGF+Jasp+Tax treatment groups. (**F**) Kcps values of the control group, EGF, EGF+Jasp, EGF+Tax and EGF+Jasp+Tax treatment groups. Data are the mean ± SEM of at least three separate experiments.

A GFAP-cpst-GFAP probe was constructed and transfected into A549 cells by a previously reported method 20-22, revealing that GFAP tension and cytoplasmic osmotic pressure increase upon EGF treatment (Fig. 3A,B). Furthermore, EGF treatment significantly increases the proportion of cytoplasmic protein particles with sizes less than 100 nm.

Then, the MF stabilizer Jasp and MT stabilizer Tax were used, revealing that MF stabilization rather than MT stabilization lessens the increase in osmotic pressure and change in nanoparticle size distribution (Fig. 3E,F and sFig. 2A). Furthermore, (6-methoxyquinolinio)acetic acid ethyl ester bromide (MQAE) was used to investigate the changes in intracellular Cl^-^ concentration 31, 32. The data indicate that the increased Cl^-^ content is closely associated with the upregulation of protein nanoparticles (Fig. 3C,D).

These results indicate that NSCLC aggressiveness is accompanied by an increase in GFAP tension, which results from intracellular protein nanoparticles and ion-induced osmotic pressure in response to EGF stimulation.

### Talin is involved in the increasing GFAP tension and intracellular osmotic pressure associated with NSCLC invasion and metastasis

**Figure 4:**
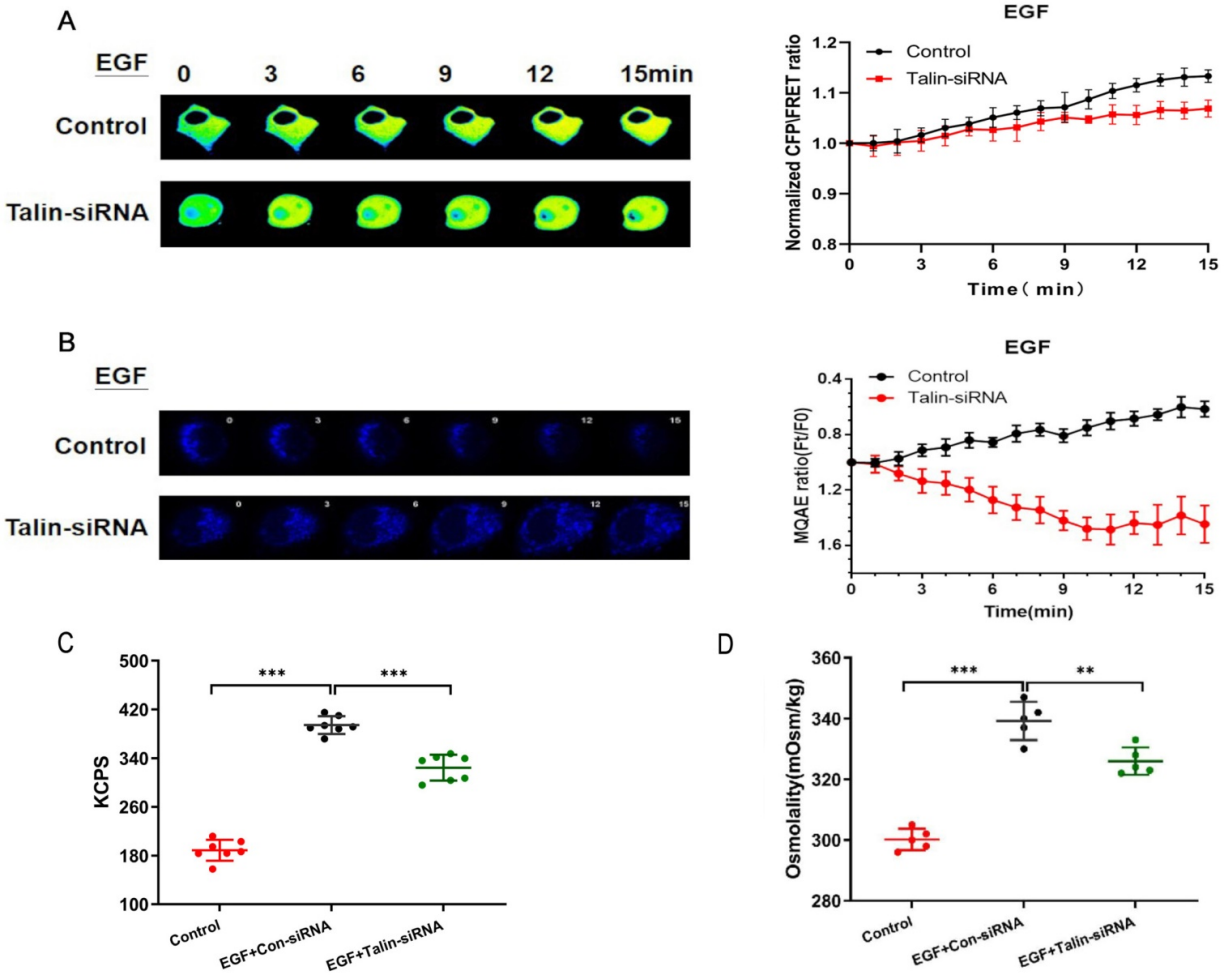
** Talin downregulation increases osmotic pressure and protein nanoparticles** (**A**) 15-min time-lapse images of FRET analysis in A549 cells expressing the GFAP-cpstFRET-GFAP probe and co-transfected with talin siRNA or control siRNA after EGF treatment. Right panel: Normalized CFP and FRET signals corresponding to GFAP force versus time. (**B**) 15-min time-lapse images of MQAE analysis in A549 cells transfected with talin siRNA or control siRNA after EGF treatment. (**C**) Kcps values of the control and talin-siRNA groups. (**D**) Osmolalities of the control and talin-siRNA group (n ≥ 3).

Talin-siRNA and the GFAP-cpst-GFAP probe were co-transfected into A549 cells in order to investigate whether talin regulates GFAP tension. We found that talin downregulation decreases GFAP tension after EGF treatment (Fig. 4A). Furthermore, talin downregulation significantly decreases Cl^-^ content (Fig. 4B) and osmotic pressure (Fig. 4C), accompanied by a decrease in count rate (Kcps) (Fig. 4D). These results indicate that talin is involved in the increased GFAP tension and intracellular osmotic pressure in NSCLC invasion and metastasis.

### Integrin activation results in talin tension and talin-related osmotic pressure in NSCLC invasion

**Figure 5:**
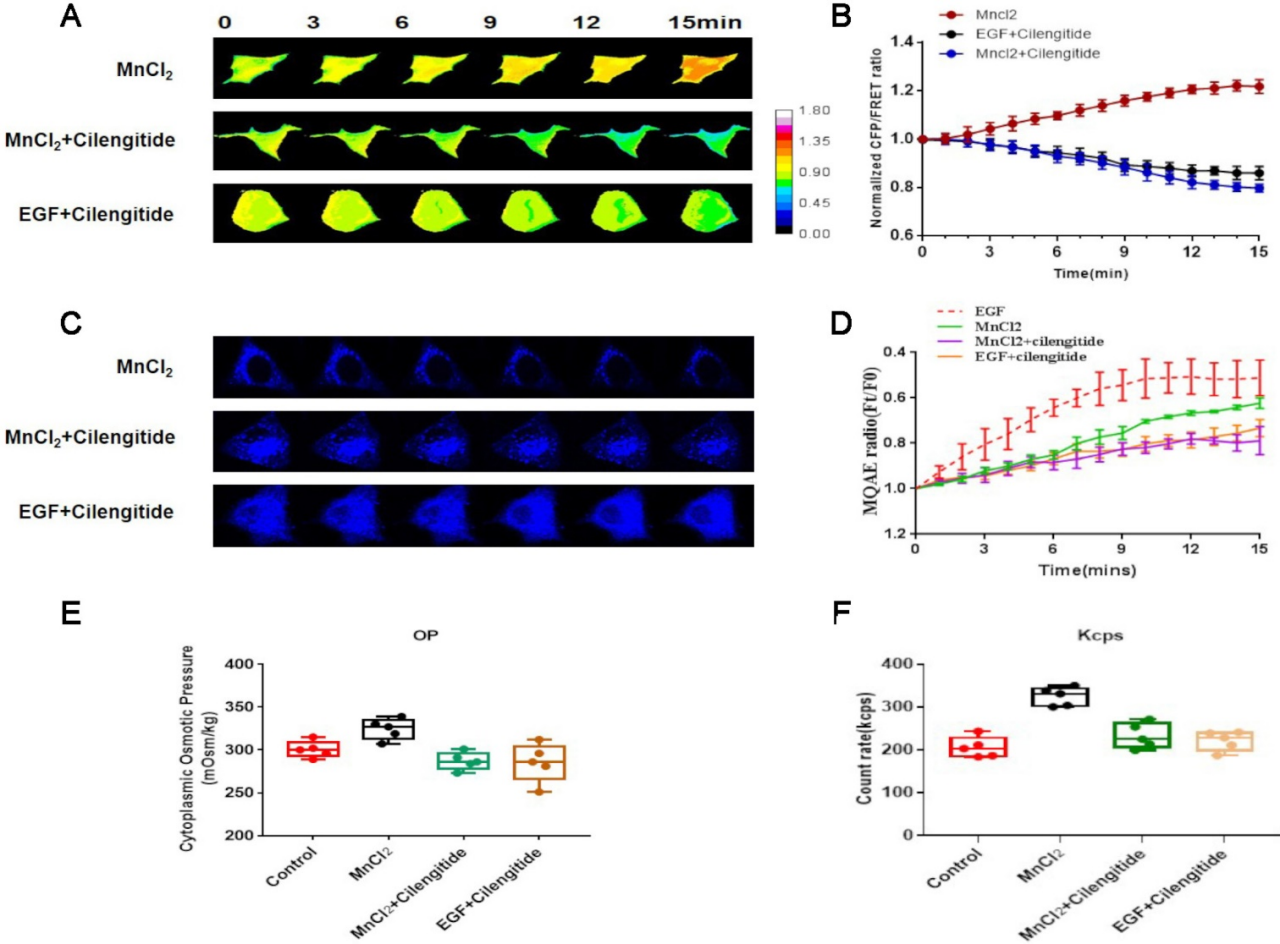
** Integrin regulates talin tension and talin-related osmotic pressure in NSCLC invasion** (**A**) 15-min time-lapse images of FRET analysis in A549 cells expressing talin-M-cpstFRET probe after MnCl_2_, MnCl_2_+Cilengitide, or Cilengitide+EGF treatments. (**B**) Normalized CFP and FRET signals corresponding to talin tension versus time. (**C**) 15-min time-lapse images of MQAE analysis after MnCl_2_, MnCl_2_+Cilengitide, and Cilengitide+EGF treatments in A549 cells. (**D**) Means of normalized MQAE fluorescence intensity. Scale bar, 10 µm. (**E**) Osmolalities of the control group and wild-type A549 cells treated with MnCl_2_, MnCl_2_+Cilengitide, or Cilengitide+EGF. (**F**) Kcps values of the control, MnCl_2_, MnCl_2_+Cilengitide, and Cilengitide+EGF treatment groups. Data are the mean ± SEM of at least three separate experiments.

Talin is an integrin-binding adaptor protein and the main mediator of integrin activation. The integrin agonist MnCl2 or its inhibitor cilengitide was co-administered with EGF to A549 cells, and subsequent FRET analysis indicated that MnCl_2_ enhances talin tension after 15-min treatment. Furthermore, 15-min time-lapse imaging showed a decreasing trend in talin tension after inhibition of integrin by cilengitide, and MnCl_2_ co-administration with cilengitide does not reverse this trend (Fig. 5A,B).

To verify whether integrin activation mediates NSCLC aggressiveness by regulating osmotic pressure, the osmolality in A549 cells was measured after MnCl_2_ or cilengitide treatment. The results indicate that integrin activation increases cytoplasmic osmotic pressure and Cl^-^ concentration. Furthermore, the upward trend in cytoplasmic osmotic pressure and Cl^-^ concentration caused by MnCl_2_ is decreased after cilengitide treatment (Fig. 5C,D,E,F).

The size distribution of cytoplasmic particles was found to be a key influencing factor of osmotic pressure. As the intracellular osmotic pressure increases, the number of cytoplasmic protein particles less than 100 nm in size is significantly increased after MnCl_2_ treatment. Much like osmotic pressure, the change in the size distribution of cytoplasmic particles is adjusted by cilengitide (sFig. 2B). These results indicate that talin tension and protein-nanoparticle-induced osmotic pressure promote A549 cell aggressiveness in response to integrin activation.

### Talin-related mechanical activity in NSCLC aggressiveness depends on connection with actin

We have demonstrated that the increase in talin tension results from inward MF force. To further investigate whether a connection between talin and actin mediates NSCLC invasion and metastasis, we designed a talin-truncated FRET probe (Talin-ABS), in which the actin binding domain is deleted, preventing actin-talin binding 21.

**Figure 6:**
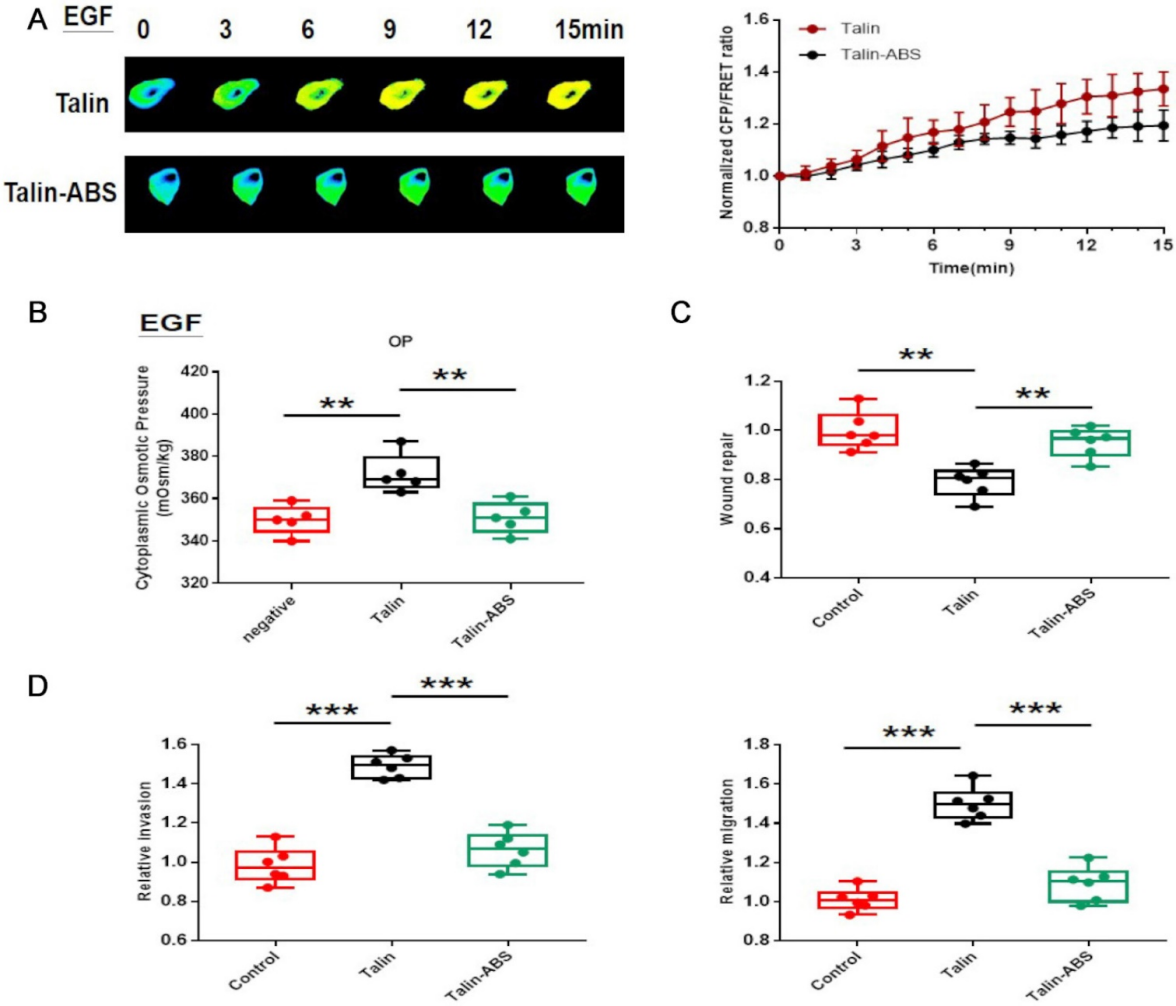
** Talin-related mechanical activity depends on connection with actin in NSCLC aggressiveness** (**A**) Left panel: 15-min time-lapse images of FRET analysis in A549 cells expressing the talin-M-cpstFRET probe and talin-ABS-M-cpstFRET after EGF treatment. Right panel: Normalized CFP and FRET signals corresponding to talin tension versus time. (**B**) Osmolalities of the control, talin, and talin-ABS groups after EGF treatment. (**C**) Normalized quantitative analysis of the wound closure rate in A549 cells transfected with talin plasmid or talin-ABS plasmid. (D) Quantitative analysis of migration and invasion rates. mean ± SEM, n ≥ 3. **P <0.01 compared with the control group, ***P <0.001 compared with the control group.

The talin tension increases more slowly after deletion of the actin binding domain in the short-term aggressive EGF model (Fig. 6A). Furthermore, actin-binding-domain deletion decreases intracellular osmotic pressure (Fig. 6B) and decreases A549 cell invasion and metastasis (Fig. 6C,D; sFig. 1C,D). These results indicate that talin-related mechanical activity promotes NSCLC invasion and metastasis, and that it depends on the interaction between talin and MF.

## Discussion

Talin/integrin signaling plays an important role in tumor progression as a convergency of chemical and mechanical signals 33-35. In the present study, the integrin receptor talin was found to be involved in NSCLC invasion and metastasis via intracellular tension activity. Talin tension and talin-related osmotic pressure promote NSCLC invasion and migration, which is regulated by MF force and integrin activity.

As a cytoskeletal protein, talin only transmits tension and cannot produce force 28. Previous studies and the present results indicate that MF force acts as the major modulator of talin tension 20, 21. The motor protein myosin, which generates an inward contraction of MFs, is the main mechanical source for tumor invasion 36, 37. Talin plays a transitive role in transmitting MF force to the cell membrane (Fig. 2). The actin-binding site 3 in the rod domain is the most important site for such activity. Deletion of actin-binding site 3 in talin blocks the traction of MFs, which reduces cell mobility 38 and thus inhibits tumor invasion and migration, indicating that inward talin tension is necessary for tumor aggressiveness (Fig. 6). Similarly, cellular blebbing, caused by local alterations in cell surface tension, has been shown to increase the invasiveness of cancer cells 39. Disruption of the interaction between talin and MFs reduces the pulling force on the cell surface and decrease blebbing in tumor cells.

In a previous study, the talin-integrin complex was found to link the extracellular matrix and promote focal adhesion formation in tumor progression 10-14. However, the results of the present study indicate that intracellular mechanical activity caused by talin is more important for cancer invasion and migration (Fig. 5 and sFig. 1). Integrins can connect talin to focal adhesion proteins, allowing the MF force to act on the extracellular matrix and promote directional movement of cancer cells. Furthermore, the downregulation of integrin activity leads to a weakened connection between the cell membrane and the microfilaments, preventing the inward MF force being translated into an applied force on the extracellular matrix.

Cell osmotic pressure is considered to be another driving force in cancer invasion and migration 29. The present data show that high-aggressiveness A549 cells exhibit integrin-related high osmolality. According to the Donnan effect 40-42 and previous studies 20-22, the increase in osmotic pressure is mainly caused by protein nanoparticle production, and depolymerization of MFs and MTs elicited by cofilin and stathmin activation 43-45. The cofilin and stathmin incise the cytoskeleton into protein nanoparticles, which absorb dissociative cations such as K+ and Na+. This absorption results in an imbalance of dissociative cations across the cell membrane causing cations to flow into cells and subsequent anion influx to balance the increasing cation presence inside the cells. This hypothesis is supported by the present results, specifically that talin/integrin activation is involved in protein nanoparticle generation and ion influx during NSCLC metastasis (Fig. 3).

Talin not only transmits MF force, it also participates in depolymerization of the cytoskeleton, increasing osmotic pressure, and thus to tumor invasion and metastasis (Fig. 4 and sFig. 1). Talin-transmitted force induces blebbing and integrin-related osmotic pressure provides an outward driving force to this blebbing. According the squeezing model 28, these two mechanical activities could synergistically contribute to the intensity of pressure in the blebbing during NSCLC invasion and migration.

In summary, the present study has identified talin as a critical regulator in NSLCL invasion and migration. Talin transmits MF force and produces osmotic pressure to increase the intensity of osmotic pressure, which is regulated by integrin activity. Higher cell aggressiveness is accompanied by increased talin tension and osmotic pressure resulting from production of intracellular protein nanoparticles.

Importantly, the use of FRET tension probes allowed us to better explore the magnitude and direction of intracellular tension in cancer cells, revealing that talin-related mechanical activity plays a regulatory role in tumor invasion. We thus suggest that talin-related inward tension and outward osmotic pressure may be potential targets for NSCLC therapy.
